# Differential effects of hydrocortisone, prednisone, and dexamethasone on hormonal and pharmacokinetic profiles: a pilot study in children with congenital adrenal hyperplasia

**DOI:** 10.1186/s13633-016-0035-5

**Published:** 2016-09-26

**Authors:** Todd D. Nebesio, Jamie L. Renbarger, Zeina M. Nabhan, Sydney E. Ross, James E. Slaven, Lang Li, Emily C. Walvoord, Erica A. Eugster

**Affiliations:** 1 Department of Pediatrics, Division of Pediatric Endocrinology/Diabetology, Indiana University School of Medicine, 705 Riley Hospital Drive, Room 5960, Indianapolis, IN 46202 USA; 2 Department of Pediatrics, Division of Pediatric Hematology/Oncology, Indiana University School of Medicine, Indianapolis, IN USA; 3 Department of Medicine, Division of Clinical Pharmacology, Indiana University School of Medicine, Indianapolis, IN USA; 4 Department of Biostatistics, Indiana University School of Medicine, Indianapolis, IN USA; 5 Department of Medical and Molecular Genetics, Computational Biology and Bioinformatics, Indiana University School of Medicine, Indianapolis, IN USA

**Keywords:** Congenital adrenal hyperplasia, Hydrocortisone, Prednisone, Dexamethasone, Pharmacogenetics

## Abstract

**Background:**

Little is known about the comparative effects of different glucocorticoids on the adrenal and growth hormone (GH) axes in children with congenital adrenal hyperplasia (CAH). We sought to compare the effects of hydrocortisone (HC), prednisone (PDN), and dexamethasone (DEX) in children with classic CAH and to investigate a potential role of pharmacogenetics.

**Methods:**

Subjects were randomly assigned to three sequential 6-week courses of HC, PDN, and DEX, each followed by evaluation of adrenal hormones, IGF-1, GH, and body mass index (BMI). Single nucleotide polymorphism (SNP) analysis of genes in the glucocorticoid pathway was also performed.

**Results:**

Nine prepubertal subjects aged 8.1 ± 2.3 years completed the study. Mean ACTH, androstenedione, and 17-hydroxyprogesterone (17-OHP) values were lower following the DEX arm of the study than after subjects received HC (*p* ≤ 0.016) or PDN (*p* ≤ 0.002). 17-OHP was also lower after HC than PDN (*p* < 0.001). There was no difference in IGF-1, GH, or change in BMI. SNP analysis revealed significant associations between hormone concentrations, pharmacokinetic parameters, and variants in several glucocorticoid pathway genes (*ABCB1*, *NR3C1*, *IP013*, *GLCCI1*).

**Conclusions:**

DEX resulted in marked adrenal suppression suggesting that its potency relative to hydrocortisone and prednisone was underestimated. SNPs conferred significant differences in responses between subjects. Although preliminary, these pilot data suggest that incorporating pharmacogenetics has the potential to eventually lead to targeted therapy in children with CAH.

## Background

Classic congenital adrenal hyperplasia (CAH) due to 21-hydroxylase deficiency results from an enzymatic block in the biosynthesis of cortisol and aldosterone. Treatment of classic CAH in children involves a challenging balance between androgen excess leading to bone age advancement due to under treatment with glucocorticoids and cortisol excess leading to impaired linear growth from overtreatment [1]. Hydrocortisone (HC) is the preferred glucocorticoid in children with CAH due to potential concerns of linear growth suppression associated with longer-acting and more potent glucocorticoid formulations [1, 2]. However, while experience is limited, dexamethasone (DEX) and single or multiple doses of prednisone (PDN) have been used successfully in children with CAH [3, 4].

Little is known about the comparative effects of HC, PDN, and DEX on the hypothalamic-pituitary-adrenal (HPA) and growth hormone axes in CAH. The aim of this pilot study was to compare hormonal and pharmacokinetic profiles after short-term treatment with HC, PDN, and DEX in children with CAH. Additional studies were performed to determine if variants in genes in the glucocorticoid pathway were associated with individual differences in responses to treatment.

## Methods

### Subjects and study design

Prepubertal children between the ages of 4 and 12 years with classic CAH followed at Riley Hospital for Children were eligible for enrollment. Exclusion criteria included medical problems that affect growth, absorption, or clearance of glucocorticoids and medications known to affect the absorption or clearance of glucocorticoids. The study was approved by the Institutional Review Board at Indiana University, and written informed consent from parents and assent from subjects, when appropriate, was obtained. All visits were conducted at the Indiana University General Clinical Research Center (GCRC). At the time of enrollment, a physical exam was performed, including height, weight, vital signs, and Tanner staging. A bone age radiograph was obtained if one had not been done within the past 6 months. Baseline blood tests at enrollment included 17-hydroxyprogesterone (17-OHP), androstenedione, and electrolytes.

Subjects were assigned to three sequential 6-week treatment courses arranged in random order during which they received the following medications: hydrocortisone (5 mg tablets; Pfizer, New York, NY) 15 mg/m^2^/day divided three times a day administered at 08:00, 15:00, and 21:00; prednisone (1 mg tablets; Roxane Laboratories Inc., Columbus, OH) 3 mg/m^2^/day divided twice a day administered at 08:00 and 21:00; and dexamethasone (0.5 mg/5 mL elixir; Morton Grove Pharmaceuticals Inc., Morton Grove, IL) 0.3 mg/m^2^/day administered daily at 21:00. All medications were taken orally. The bioequivalence and potency conversion of HC to PDN to DEX was estimated to be 1 to 5 to 50, based on clinical practice guidelines from the Endocrine Society regarding glucocorticoid dosing in CAH [1]. A pill cutter and oral syringe were given to each subject. Subjects remained on their usual dose of mineralocorticoid replacement throughout the study. Subjects were instructed to administer stress dose steroids by tripling the oral dose of glucocorticoid in times of illness or fever. If stress dosing was administered within a week of a scheduled GCRC admission, it was postponed until the subject had not received stress dose steroids for at least 7 days.

At the end of each 6-week period, subjects were admitted to the GCRC for 25 h. A physical exam was performed, including height, weight, vital signs, and Tanner staging. A peripheral IV was placed from which all blood samples were obtained. At 08:00, sodium, potassium, IGF-1, 17-OHP, and androstenedione were measured. For those taking HC, cortisol was measured before the 08:00 dose and then at 20, 40, 60, 80, 120, and 240 min in order to evaluate cortisol metabolism and clearance. While on PDN, cortisol was measured as a surrogate for PDN metabolism and clearance. When patients were on DEX, a dexamethasone level was drawn at 20:00 before the 21:00 DEX dose was administered. Measurements of 17-OHP were obtained every 2 h (13 total per each overnight stay); growth hormone every hour from 20:00 to 08:00 (13 total per each overnight stay); adrenocorticotropic hormone (ACTH) at 14:00 and then every 2 h from 00:00 to 06:00 (5 total per each overnight stay); and androstenedione at 20:00 (2 total per each overnight stay). At the first GCRC admission, 5 mL of blood was drawn and frozen in the GCRC Pharmacogenetic Core Lab to be used for DNA extraction.

All subjects were contacted by telephone two weeks after starting a new glucocorticoid regimen to review administration and compliance. Pill counts and determination of elixir volumes were performed at GCRC admissions to assess compliance. At the end of the 18-week study, subjects returned to their pre-study glucocorticoid regimen.

### Laboratory assays

All blood samples were analyzed at the GCRC, except for genomics and dexamethasone samples which were analyzed at Esoterix (Calabasas Hills, CA) by high performance liquid chromatography (HPLC) tandem mass spectrometry. Standard clinical laboratory methods were used to measure serum electrolytes. 17-OHP was measured by radioimmunoassay (RIA) (MP Biomedicals, Orangeburg, NY). The following hormones were measured by enzyme-linked immunosorbent assay (ELISA): cortisol, androstenedione, ACTH, IGF-1 (ALPCO Diagnostics, Salem, NH) and growth hormone (Alpha Diagnostic, Inc., San Antonio, TX).

DNA was extracted with QIAGEN QIAamp® DNA Blood Mini and Maxi kits (QIAGEN, Valencia, CA) and genotyped for single nucleotide polymorphisms (SNPs) in genes in the glucocorticoid pathway as previously described [5]. SNPs analyzed included those in the following genes: importin 13 or *IPO13* (rs6671164, rs4448553, rs1990150, rs2240447, rs2486014, rs2301993, rs7412307, rs2428953), nuclear receptor subfamily 3, group C, member 1 or *NR3C1* (rs41423247), glucocorticoid-induced transcript 1 or *GLCCI1* (rs37973), ATP-binding cassette, subfamily B, member 1 or *ABCB1* (rs1045642, rs2032582, rs1128503), cytochrome P450, subfamily IIIA, polypeptide 7 or *CYP3A7* (rs2257401, rs2687133), stress-induced phosphoprotein 1 or *STIP1* (rs2236647), low density lipoprotein, oxidized, receptor 1 or *OLR1* (rs3736233), adenylate cyclase 2 or *ADCY2* (rs2230739), corticotropin-releasing hormone receptor 1 or *CRHR1* (rs242941, rs1876828), and Fc fragment of IgE, low affinity II, receptor for or *FCER2* (rs28364072).

### Statistical analysis

Statistical analyses were performed to determine if there was an association between the three glucocorticoids and biochemical and anthropometric outcomes. These outcomes were measured over time, and thus each participant had multiple measures for each outcome. Generalized linear models were used to analyze the data in order to account for repeated measures and to ensure that the proper covariance structure was used. Pairwise comparisons were also performed using a Bonferroni correction. Overall analyses were considered significant at *p* ≤ 0.05 and pairwise comparisons among the three drugs were considered significant at *p* ≤ 0.017. All analytic assumptions were verified and log transformed when the data did not follow the normal distribution.

For 17-OHP, additional analyses were performed to determine the proportion of time spent within the predetermined target range of 100–1000 ng/dL. Each time point was connected with a straight line to its next neighbor and integration methods were performed to derive the total area and specified target areas. ANOVA models were used to determine if there was a significant difference in the proportion of time within the target range between the study drugs.

BMI z-scores were generated using the Center for Disease Control’s growth chart training resource. ANCOVA models were used to determine if there was a significant difference in BMI z-scores following treatment with the three different study drugs. As the glucocorticoids were administered at different combinations, this analysis was adjusted for the previous drug that the subject was on.

All analytic assumptions were verified. All analyses were performed using SAS v9.3 (SAS Institute, Cary, NC).

The pharmacokinetic measurements were based on three summaries: average, area under the concentration curve (AUC), and decreasing rate or slope. The average was calculated based on the average measurement among all sampling time points. The AUC was calculated based on the trapezoid rule, and the decreasing rate was calculated based on the measurements from the last three sampling time points.

The associations between relevant phenotypes and SNPs were assessed through three different genetic models: dominant, recessive, and additive. The dominant model compares the genotype homozygous dominant (XX) with the genotypes containing one or both recessive, or variant alleles (Xx or xx). The recessive model compares the genotype homozygous recessive (xx) versus genotypes with one or both dominant alleles (XX or Xx). An additive model examines whether each copy of an allele contributes to an additional risk, so XX would be the highest, Xx would have less risk, and xx would have no risk [6]. Linear regression was applied to analyze these genetic associations. T-tests were used to determine directionality. As this was an exploratory analysis, any association with a *p*-value ≤ 0.05 was considered statistically significant. The Bonferroni justification was not applied in the genetic data analysis. The genetic data analyses were conducted in PLINK (http://pngu.mgh.harvard.edu/purcell/plink/).

## Results

### Subjects

Nine subjects with classic CAH (8 with salt-wasting and 1 with simple virilizing) aged 8.1 ± 2.3 years (range: 4.8–11.6 years) completed the study. Four additional subjects were enrolled but withdrew due to difficulty maintaining peripheral IV access. One additional subject was excluded when his screening visit revealed he was pubertal. No other subjects met exclusion criteria. Patient characteristics including baseline hormonal values at study entry are summarized in Table 1. Electrolytes and vital signs were normal in all subjects.

**Table 1:** Baseline characteristics at enrollment of subjects who completed all 3 arms of the study

Number of subjects	9 (5 female)
Glucocorticoid regimen prior to entering the study	Hydrocortisone (7), prednisone (2)
Dose of hydrocortisone (mg/m2/day) equivalent	15.6 ± 4.0
Age (years)	8.1 ± 2.3
Bone age (years)	8.6 ± 2.9
Height (cm); z-score	127.6 ± 17.9; 0.0 ± 1.2
Weight (kg); z-score	39.1 ± 24.0; 1.0 ± 1.4
BMI (kg/m^2^); z-score	22.0 ± 8.1; 1.2 ± 1.1
17-hydroxyprogesterone (ng/dL)	991.1 ± 978.5 89.4 (0.4–2500.0)
Androstenedione (ng/dL)	35.2 ± 22.5 22.3 (2.0–392.0)

Values are mean ± standard deviation, with 17-hydroxyprogesterone and androstenedione also including median (minimum – maximum) due to data skewness

### Hormonal studies and BMI z-scores

**Fig. 1:**
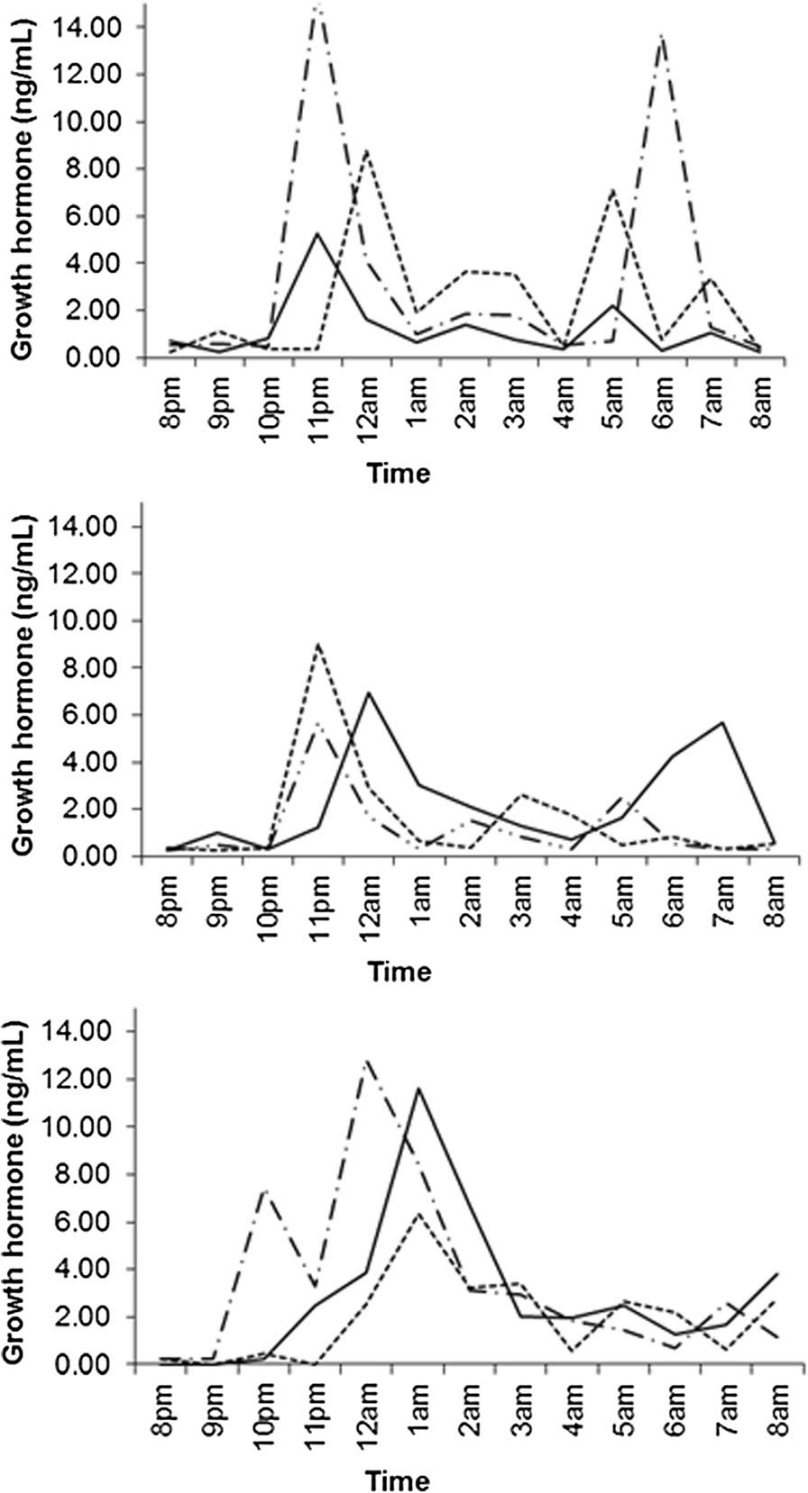
Representative GH profiles in three subjects. There is normal variability and pulsatility of GH secretion for each glucocorticoid (solid line for PDN, solid-dash line for HC, and dashed line for DEX)

Mean ACTH, androstenedione, and 17-OHP values were all significantly lower following treatment with DEX when compared to HC (*p* = 0.016, *p* = 0.016, *p* < 0.001) and PDN (*p* < 0.001, *p* = 0.002, *p* < 0.001). Interestingly, 17-OHP levels were significantly lower following treatment with HC than on PDN (*p* < 0.001). No other differences in adrenal hormones between treatment groups were seen. IGF-1 levels did not differ among the three treatment groups (*p* = 0.980), nor did GH levels (*p* = 0.127), or change in BMI z-scores (*p* = 0.648). Mean IGF-1 SDS (adjusted for age, gender, and Tanner stage) also did not differ for each glucocorticoid (*p* = 0.957): HC (1.57), PDN (1.61), and DEX (1.78). Representative GH profiles showing preserved GH pulsatility from three of the subjects are shown in Fig. 1. None of the subjects exhibited clinical signs of glucocorticoid excess during the study. The significant differences in hormone levels between treatment pairs are summarized in Table 2.

**Table 2:** Summary of differences of log transformed data between treatment pairs

Drug pair	ACTH		Androstenedione		17-OHP	
	Difference (95 % CI)	*p*-value	Difference (95 % CI)	*p*-value	Difference (95 % CI)	*p*-value
DEX-HC	−0.55 (−0.99, −0.12)	0.016**	−0.64 (−1.15, −0.14)	0.016**	−1.59 (−1.94, −1.23)	< 0.001**
DEX-PDN	−0.90 (−1.33, −0.47)	< 0.001**	−0.90 (−1.40, −0.40)	0.002**	−2.44 (−2.80, −2.09)	< 0.001**
HC- PDN	−0.35 (−0.78, 0.09)	0.110	−0.26 (−0.76, 0.25)	0.293	−0.86 (−1.21, −0.50)	< 0.001**

Values are expressed as the difference between drug groups (first drug minus the second drug) with 95 % confidence interval (CI)

** Significant *p*-value is ≤ 0.017

**Fig. 2:**
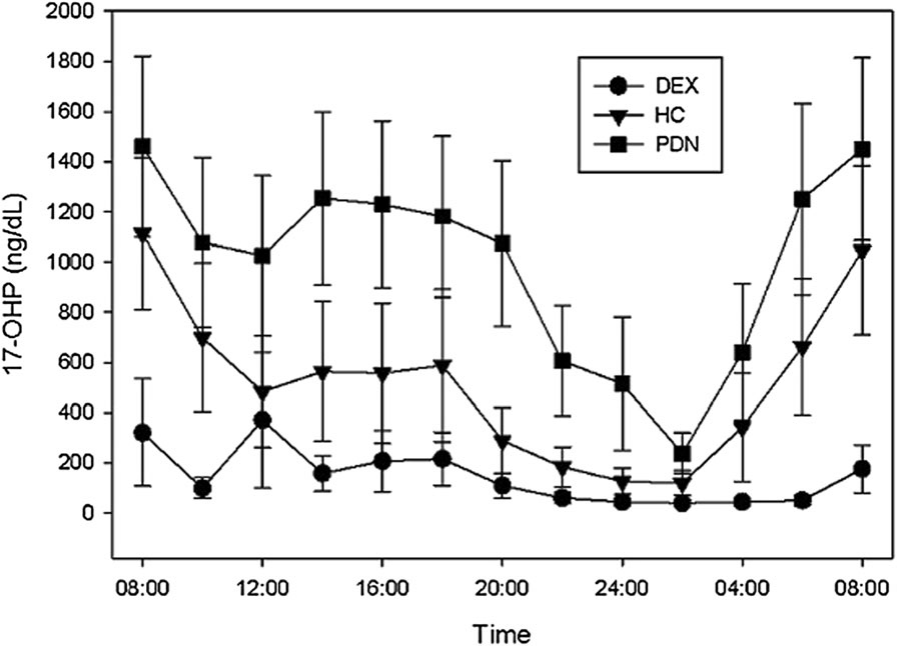
Mean 17-hydroxyprogesterone (17-OHP) concentration by time points. 17-OHP was measured every 2 h during each inpatient stay from 08:00 to 08:00 the next day. 17-OHP was lowest on DEX and highest on PDN at each time point (*p* < 0.001). Bars represent the 95 % confidence interval for the standard error

Mean 17-OHP values were lowest on DEX and highest on PDN at each measured time point (Fig. 2). The majority of 17-OHP values during treatment with each glucocorticoid were outside of the target range (75 % for DEX, 70 % for HC, and 80 % for PDN) and did not differ between groups (*p* = 0.714).

### Dexamethasone levels

Eight subjects had trough DEX levels of < 30 ng/dL. Only one subject had a detectable DEX level of 56 ng/dL, which corresponded to low 17-OHP and androstenedione concentrations. However, 17-OHP measurements in three subjects with undetectable DEX levels were nearly all < 100 ng/dL and androstenedione levels were also low.

### Pharmacokinetics and pharmacogenetics

**Fig. 3:**
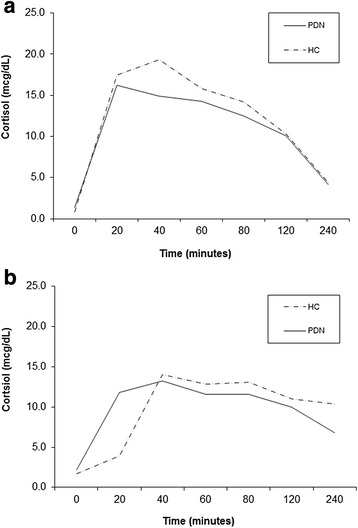
Variable cortisol pharmacokinetics were noted in subjects taking hydrocortisone (HC) and prednisone (PDN). For example, the subject in (**a**) metabolized and cleared glucocorticoids faster than the subject in (**b**)

**Fig. 4:**
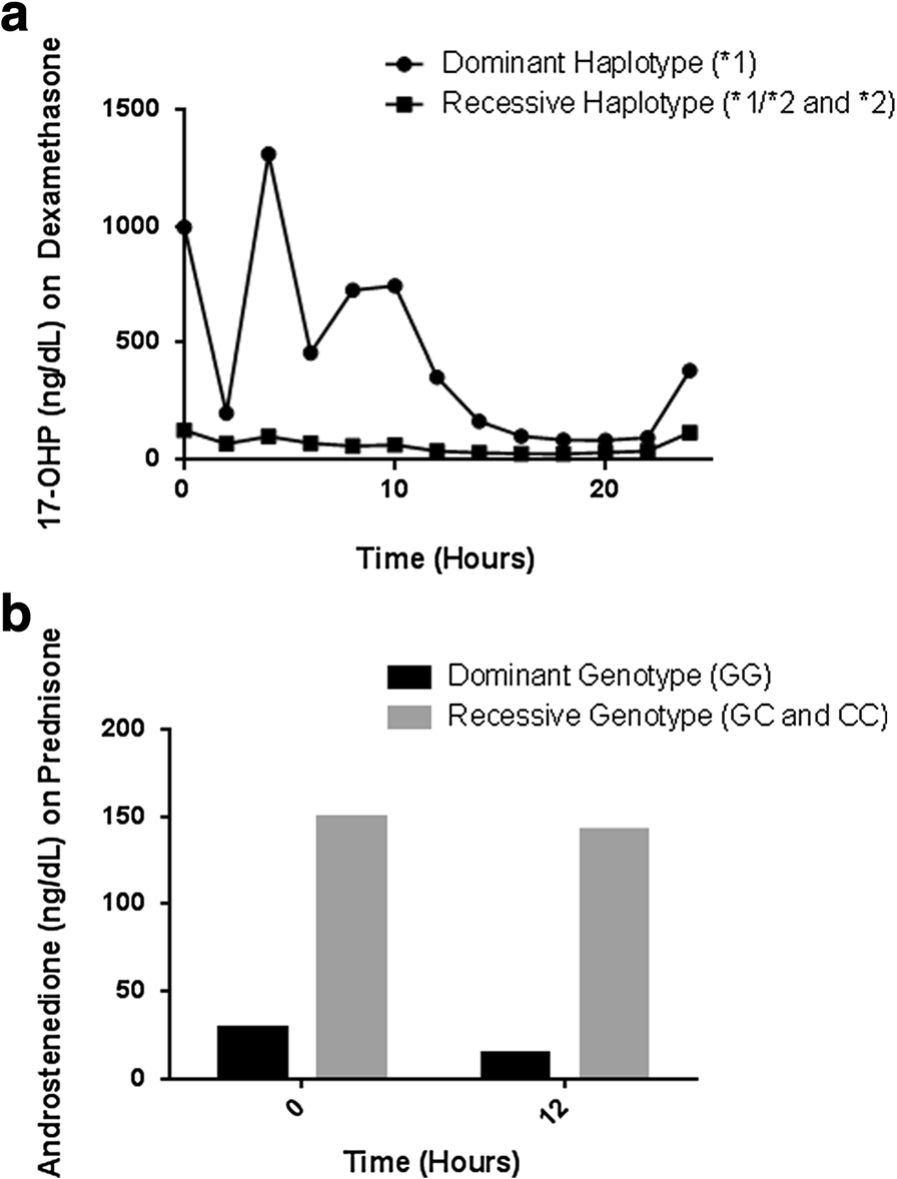
In **a**, when comparing the ABCB1 *1 dominant haplotype with the ABCB1 recessive haplotypes *1/*2 and *2, the subjects homozygous for the *1 haplotype had less suppression of 17-OHP on DEX compared to the others. In **b**, those expressing the dominant genotype had greater suppression of androstenedione on PDN than the recessive genotype

As illustrated in Fig. 3, there was variability in glucocorticoid metabolism and pharmacokinetics between subjects. Due to the small sample size, data analysis is reported using dominant genetic models, which examine the associations between carrying the dominant allele for each SNP and downstream pharmacokinetic effects. Based on the dominant model analyses, gene variants were correlated with hormone concentrations. First, for *ABCB1* (rs2032582 and rs1045642), subjects carrying at least one copy of the variant (GA or AA) had significantly lower concentrations of 17-OHP on the DEX arm as compared to subjects with the homozygous wild-type (GG) genotype (*p* = 0.025 for both). Showing a similar trend for *ABCB1* (rs1128503) when on DEX, subjects carrying at least one variant allele (CA or AA) had significantly lower concentrations of 17-OHP compared to homozygous wild-type (CC) subjects (*p* = 0.025). For *NR3C1* (rs41423247), subjects with at least one copy of the variant allele (GC or CC) had significantly higher concentrations of androstenedione when on PDN as compared to homozygous wild-type (GG) subjects (*p* = 0.004). There were no significant genetic associations identified when subjects were on HC. These results are shown in Table 3 and Fig. 4.

**Table 3:** Average association analysis using a dominant model of genetic variants and their effect on hormone levels during glucocorticoid treatments

Hormone	Drug	Gene	CHR	SNP	Basepair	Minor allele	N	*P* value
Androstenedione	Prednisone	NR3C1	5	rs41423247	142778575	C	8	0.004
17-OHP	Dexamethasone	ABCB1	7	rs1045642	87138645	A	9	0.025
17-OHP	Dexamethasone	ABCB1	7	rs2032582	87160618	A	9	0.025
17-OHP	Dexamethasone	ABCB1	7	rs1128503	87179601	A	9	0.025

*Abbreviations*: *CHR* chromosome, *SNP* single nucleotide polymorphism, *N* number, *17-OHP* 17-hydroxyprogesterone, *NR3C1* nuclear receptor subfamily 3 group C member 1, *ABCB1* ATP-binding cassette sub-family B

To investigate total drug exposure over time, area under the concentration curve (AUC) analysis was performed, with significant associations noted only during the PDN arm of the study (Table 4). For importin-13 (*IPO13*, rs6671164 and rs7412307), subjects homozygous for the variant allele had the least exposure to glucocorticoid followed by those who were heterozygous for the variant followed by those homozygous for the wild-type (*p* = 0.015 for all). Inversely, for *IPO13* variants rs4448553, rs1990150, rs2240447, rs2301993, and rs2428953, subjects who were homozygous for the variant allele had the most exposure to glucocorticoid followed by those who were homozygous for the wild type followed by subjects who were heterozygous for the variant (*p* ≤ 0.023 for all). Lastly, subjects who were heterozygous for the variant allele for the glucocorticoid induced transcript 1 (*GLCCI1*, rs37973) had the least exposure to glucocorticoid followed by those homozygous for the wild-type followed by subjects who were homozygous for the variant allele (*p* = 0.037 for all). There were no significant correlations identified for these gene variants when subjects were on HC or DEX.

**Table 4:** Area under the curve and slope analysis of cortisol for genetic variants

Drug	Gene	CHR	SNP	Basepair	Minor allele	N	*P* value
Area under the curve analysis:							
Prednisone	IPO13	1	rs6671164	44403489	G	8	0.015
Prednisone	IPO13	1	rs4448553	44411589	A	9	0.007
Prednisone	IPO13	1	rs1990150	44414127	G	9	0.023
Prednisone	IPO13	1	rs2240447	44415415	C	9	0.007
Prednisone	IPO13	1	rs2301993	44426025	C	9	0.007
Prednisone	IPO13	1	rs7412307	44433864	G	8	0.015
Prednisone	IPO13	1	rs2428953	44443459	A	9	0.023
Prednisone	GLCCI1	7	rs37973	8007876	G	9	0.038
Slope analysis:							
Hydrocortisone	ABCB1	7	rs1045642	87138645	A	9	0.026
Hydrocortisone	ABCBI	7	rs2032582	87160618	A	9	0.026
Hydrocortisone	ABCB1	7	rs1128503	87179601	A	9	0.026

*Abbreviations*: *CHR* chromosome, *SNP* single nucleotide polymorphism, *IPO13* importin-13, *GLCCI1* glucocorticoid-induced transcript 1, *ABCB1* ATP-binding cassette sub-family B member 1

Slope analysis was performed to determine cortisol clearance (Table 4). Using the dominance model, subjects homozygous for *ABCB1* variants (rs1045642, rs2032582, and rs1128503) had faster clearance of cortisol while on HC as compared to homozygous wild-type subjects (*p* = 0.026, *p* = 0.026, and *p* = 0.026, respectively). There were no significant correlations between gene variants and cortisol clearance for subjects on PDN.

## Discussion

This pilot study compared the hormonal effects and pharmacokinetic profiles of HC, PDN, and DEX in nine prepubertal children with classic CAH. We used a potency conversion between HC to PDN to DEX of 1 to 5 to 50. However, our data demonstrate that DEX was more potent than both HC and PDN as children on the DEX arm had significantly lower adrenal hormone levels compared to those on either HC or PDN. Although glucocorticoid equivalencies are debatable, our findings are in line with other studies suggesting that DEX is at least 70 times [3] and up to 80 to 100 times or more [7–9] potent than HC in regards to suppressing adrenal androgen production. Unlike for DEX, we did not find any difference in ACTH or androstenedione concentrations during treatment with HC and PDN. In contrast, 17-OHP values were significantly higher when children were receiving PDN than when they were treated with HC. This suggests that PDN may be ≤ 5 fold more potent than HC and conflicts with other reports suggesting that PDN is 10 to 15 times more potent than HC [9, 10]. It is important to note that these findings may not reflect what is seen with prednisolone, which is the active metabolite of PDN.

Laboratory monitoring during glucocorticoid therapy in children with CAH is difficult since hormone concentrations can vary widely depending on the time of day and most recent glucocorticoid dose [11]. By measuring frequent hormone levels during each inpatient stay, we were able to characterize the degree of hormone variability. In particular, there were wide fluctuations in 17-OHP levels within individual subjects demonstrating that a single 17-OHP measurement is problematic in terms of reflecting the degree of biochemical control. This marked variability in 17-OHP has been attributed to circadian patterns [12, 13] and exaggerated responses to stress [14, 15]. Androstenedione also has a circadian rhythm but the magnitude of variability is less than that seen with 17-OHP [16] and levels often correlate better with overall CAH control [17]. Although not routinely used in the management of CAH, ACTH levels have been shown to be a useful adjunct [18], and as seen in our study, often correlate with other hormone markers of biochemical control.

Nightly dosing of DEX can lead to overtreatment with greater suppression of early morning adrenal steroid concentrations [19, 20]. All but one of our subjects had an undetectable trough DEX level, but many of our subjects on DEX had low 17-OHP and androstenedione levels. This implies that trough DEX levels do not accurately reflect the biological effect at a tissue or cellular level. Despite disparities in adrenal steroids, there was no difference in GH, IGF-1, or BMI during treatment with the three glucocorticoids. However, given the short duration of our study, any presumption regarding the implication of this observation on long-term growth would be premature.

Very few studies, also with small sample sizes, have compared different glucocorticoids in either children or adults with CAH [7, 20–22], and ours is the only one to compare three different preparations in a prepubertal cohort. While overall results have been conflicting, other investigators have also noted a greater degree of suppression than expected with DEX [7], as was the case in our study. In our study, significant correlations were found between *ABCB1*, *NR3C1*, *IPO13* and *GLCCI1* genotypes and hormone concentrations and/or clearance. While such findings could be spurious, it is intuitive that differences in glucocorticoid-related genes could impact individual physiologic responses. The *ABCB1* gene encodes for P-glycoprotein, which is a transmembrane transporter that acts as a cellular drug efflux pump for various substances, including glucocorticoids [23]. Clinical response to glucocorticoids has been shown to be influenced by individual SNPs as well as haplotypes in *ABCB1* [24, 25]. For example, polymorphisms in *ABCB1* have been associated with steroid resistance in children with nephrotic syndrome [26, 27] and steroid response in those with inflammatory bowel disease [28, 29]. In our study, subjects with specific *ABCB1* genetic variants were found to metabolize and clear cortisol faster when on HC. Polymorphisms in *ABCB1* and other relevant genes might explain why some patients with CAH require unexpectedly low or high doses of glucocorticoids to achieve optimal biochemical control, and preemptive genetic testing may facilitate selection of a more appropriate starting dose for individual patients. Furthermore, experimental regimens and novel medical therapies currently under investigation may eventually make a one glucocorticoid approach obsolete in the treatment of CAH.

The *NR3C1* gene encodes for the glucocorticoid receptor (GR). Other research has shown that individuals with the *Bcl*I (rs41423247) polymorphism have increased sensitivity and responsiveness to glucocorticoids [30, 31]. This SNP has also been associated with increased BMI, hypertension, and cardiovascular disease in the general population [32]. Adults with CAH who possess the *Bcl*I polymorphism have also been found to have a higher BMI, waist circumference, and systolic blood pressure [33] but this has not been found in children [34]. We did not evaluate for correlations with BMI in our subjects with this SNP given the small size of our population. However, we did find significant associations between the rs41423247 variant and androstenedione concentrations when subjects were on PDN.

Subjects in our study with genetic variants in *IPO13* and *GLCCI1* had variable exposure to glucocorticoids when on PDN. Both *IPO13* and *GLCCI1* genotypes are associated with glucocorticoid exposure in children on PDN [35, 36]. *IPO13* regulates the nuclear translocation of the GR. Polymorphisms of *IPO13* have been associated with airway hyper-responsiveness and reactivity in children with asthma, suggesting that *IPO13* variation might improve endogenous glucocorticoid bioavailability in the cell nucleus [35]. The function of *GLCCI1* is not well understood. SNPs in *GLCCI1* in patients with asthma have been associated with a decreased response to inhaled glucocorticoids [36]. However, differences in gene variants of *GLCCI1* have not been shown to be significant in other diseases, such as steroid resistant nephrotic syndrome [37].

Limitations of our pilot study include the small sample size, lack of a washout period, previous treatment with different glucocorticoids, and short exposure to each glucocorticoid. Although significant pharmacogenetic correlations were detected, it is premature to draw firm conclusions about the possible functional role of these genetic variants [38]. Another limitation is that hormone levels were measured using RIA and ELISA instead of more precise techniques, such as liquid chromatography and tandem mass spectrometry; however, this is similar to the clinical setting. Finally, we acknowledge that the potency conversion used in our study is open to debate. Determining equivalent glucocorticoid dosing is complex, varies between individuals and can be based on anti-inflammatory effect, growth-retarding effect, and/or androgen-suppressive effect. For example, while DEX may be up to 80 times more potent than cortisol for growth-retarding effect, it appears to be only 30 times more potent than cortisol in regards to anti-inflammatory effect [39]. While extremely preliminary, our findings are intriguing and warrant replication in a larger population to see if there is a true differential versus a dose or equivalency-related effect.

## Conclusions

It would be unrealistic to expect there to be one best glucocorticoid regimen for all children with classic CAH. In our subjects, DEX resulted in over dosing while PDN often lead to under dosing. During treatment with each glucocorticoid, there was a great deal of individual variability in hormone concentrations. The majority of 17-OHP values were outside of the target range, suggesting that 17-OHP measurements to guide glucocorticoid dosing are problematic at best. Given inherent differences in individual physiology, pharmacogenetics may help to predict steroid sensitivity and response in patients with CAH. Ultimately, such information could lead to more targeted dosing for individual patients resulting in improved clinical care.

## Abbreviations

ACTH: Adrenocorticotropic hormone
BMI: Body mass index
CAH: Congenital adrenal hyperplasia
DEX: Dexamethasone
GCRC: General clinical research center
GH: Growth hormone
HC: Hydrocortisone
HPA: Hypothalamic-pituitary-adrenal
PDN: Prednisone
SNP: Single nucleotide polymorphism
